# Challenges to integrating programs for the elimination of mother-to-child transmission of HIV, syphilis, and hepatitis B into antenatal care: Experiences from Indonesia

**DOI:** 10.1371/journal.pgph.0002977

**Published:** 2024-03-06

**Authors:** Luh Putu Lila Wulandari, Dinar Saurmauli Lubis, Desak Putu Yuli Kurniati, Karjono Sumintang, Dewa Ayu Mirah Ardrini, Putri Mariani, Pande Putu Januraga, Artha Camellia, Ni Made Diah Permata Laksmi, Laila Mahmudah, Jason J. Ong, Louise Causer, Marco Liverani, Rebecca Guy, Virginia Wiseman

**Affiliations:** 1 The Kirby Institute, University of New South Wales, Sydney, Australia; 2 Department of Public Health and Preventive Medicine, Faculty of Medicine, Udayana University, Bali, Indonesia; 3 Bali Provincial Health Office, Denpasar, Indonesia; 4 Center for Public Health Innovation, Faculty of Medicine, Udayana University, Bali, Indonesia; 5 UNICEF Indonesia, Jakarta, Indonesia; 6 The Indonesia Ministry of Health, Jakarta, Indonesia; 7 Melbourne Sexual Health Centre, Alfred Health, Melbourne, Australia; 8 Central Clinical School, Monash University, Melbourne, Australia; 9 China-Australia Joint Research Center for Infectious Diseases, School of Public Health, Xi’an Jiaotong University Health Science Center, Xi’an, China; 10 Department of Clinical Research, London School of Hygiene & Tropical Medicine, London, United Kingdom; 11 Department of Global Health and Development, London School of Hygiene & Tropical Medicine, London, United Kingdom; 12 Faculty of Public Health, Mahidol University, Bangkok, Thailand; 13 School of Tropical Medicine and Global Health, Nagasaki University, Nagasaki, Japan; PLOS: Public Library of Science, UNITED STATES

## Abstract

The WHO’s Asia-Pacific framework for triple elimination recommends that countries evaluate their programs for the elimination of mother-to-child transmission of HIV, syphilis, and hepatitis B (EMTCT), including identifying gaps to improve program planning and the implementation of elimination strategies in antenatal care (ANC) services. In 2022, the Indonesian Ministry of Health reported that only 39% of pregnant women were tested for HIV, 14% for syphilis, and 28% for hepatitis B, respectively. We conducted a qualitative study involving a focus group discussion (FGD) and in-depth interviews with 25 key stakeholders in Bali and West Nusa Tenggara Provinces to identify specific challenges to testing for HIV, syphilis, and hepatitis B in ANC settings. Thematic analysis was used to identify the themes generated from the data. Health system bottlenecks experienced by stakeholders included supply chain management issues involving stock forecasting and stock monitoring, stock-outs of rapid test reagents which were particularly most frequent and for longer durations for syphilis and hepatitis B, high staff turnover, lack of staff training on how to perform the test, the complexity and time needed to record the data on women’s characteristics, risk behaviours, and testing in both paper format and into the computer-based surveillance systems, discrepancies in program coverage data from different divisions of the district health office involved in the reporting system, high levels of stigma that prevented women from being followed up, challenges in notifying partners, and inadequate reporting and referral of women from private providers to public ones for testing. Interventions addressing the above challenges are worthy of consideration to improve the health system function and integrate EMTCT into the ANC settings.

## Introduction

Mother-to-child transmission of HIV, syphilis, and hepatitis B can lead to adverse pregnancy outcomes and even an increased risk of infant mortality. Without intervention, the HIV transmission rates from mothers with HIV to their babies range from 15% to 45% [1]. Infants born to mothers with HIV have more than nine times the risk of death in six weeks and more than 21 times the risk in four months compared to those born from an HIV-negative mother [2]. Between 70–90% of babies born to mothers with hepatitis B surface antigen (HBsAg) and hepatitis B e antigen (HBeAg) during pregnancy become infected with hepatitis B [3]. Syphilis infection during pregnancy can lead to various adverse pregnancy outcomes, including early foetal loss, stillbirth, prematurity, low birth weight, neonatal and infant death, and congenital syphilis among newborns [4].

In 2020, the WHO reported that Indonesia ranked fifth in the Asia-Pacific region for the prevalence of syphilis among antenatal care (ANC) attendees (3.2%) and highest for HIV among pregnant women (0.7%) [5]. Data from the Indonesian Ministry of Health (MoH) has also reported a high prevalence of hepatitis B among pregnant women. In 2019, 1.8% of pregnant women in Indonesia tested positive for hepatitis B; in some provinces, the rate was higher than 5% [6].

HIV and syphilis testing rates in ANC settings in Indonesia were the lowest among the ten priority countries identified by the WHO for urgent intervention to reach the elimination of mother-to-child transmission (EMTCT) of HIV, syphilis, and hepatitis B [7]. Despite having an ANC attendance rate of 88% [8], only 39% of pregnant women were tested for HIV, 14% for syphilis, and 28% for hepatitis B, respectively in the period from January to August 2022 [9]. In addition, only 29%, 47%, and 35% of women who tested positive were treated for HIV, syphilis, and hepatitis B, respectively [9]. Consequently, the number of children born and living with these three pathogens continues to rise. From the latest available data, the rate for HIV MTCT in 2020 was 29.8%; the congenital syphilis case rate in 2016 was 1.2 per 100,000 live births; and hepatitis B prevalence among children 5–6 years in 2015 was 1.8% [10].

The WHO Regional Office for the Western Pacific developed a regional framework in 2018 for the EMTCT of HIV, syphilis, and hepatitis B in Asia and the Pacific by 2030 [11]. In keeping with this framework, the Indonesian MoH introduced its EMTCT program in 2018. This program requires pregnant women seeking ANC services at health facilities for the first time to be tested for HIV, syphilis, and hepatitis B using rapid point-of-care tests unless they explicitly opt-out [12]. Separate rapid tests are used to screen for HIV, syphilis, and hepatitis B. The MoH has also issued guidelines for healthcare staff to treat, manage delivery plans, and provide counselling and contraception planning for women who test positive for any of these three pathogens [13].

The WHO’s Asia-Pacific framework for the EMTCT recommends that countries regularly evaluate their EMTCT program, identifying gaps as well as opportunities to improve program planning and the implementation of elimination strategies for HIV, syphilis, and hepatitis B in ANC settings [11]. Intensive EMTCT program assessment, as outlined in the WHO guidelines, is a foundational requirement for EMTCT program validation by the EMTCT validation secretariats, committees, and teams at national, regional, and global levels [14]. However, to date, only 16 countries worldwide have been validated for the EMTCT of HIV, syphilis, or hepatitis B [15].

Previous studies in Indonesia examining the implementation of HIV and/or syphilis testing by ANC services in Indonesia have identified a number of challenges, including lack of district guidelines and clinic standard operating procedures (SOPs) to support the national policy for the EMTCT [16], lack of access to testing by pregnant women due to distance and the limited number of primary health care centres offering testing [17], inconvenient opening hours of testing services [18], low level of knowledge on HIV and/ or syphilis testing policies among health providers [19], poor quality counselling [20], lack of privacy and confidentiality [21], and significant variation in the level of health infrastructure needed for service delivery across geographic areas and by provider type (i.e. private/public) [22]. There have been no recent studies exploring the perspectives of policymakers and frontline health workers on challenges in the integration of the EMTCT of HIV, syphilis, and hepatitis B program into ANC services since its implementation in 2018. This evidence will help strengthen the health system and inform the design and implementation of Indonesia’s EMTCT program, thus making the program more responsive to the needs of women and newborns.

## Methods

### Context

In its 2020–2024 National Mid-Term Development Strategy, the Government of Indonesia recognised a number of challenges concerning its maternal and child health (MCH) workforce, referral systems, and the management of MCH health services [23]. In the same report, the government pledged its commitment to reducing maternal and newborn mortality, in the context of achieving the Sustainable Development Goals for maternal and newborn health by 2030 [23]. ANC services in Indonesia are delivered through the MCH program, by both public (*puskesmas* (a government-funded primary and public health care centre at the subdistrict level), their network, and government hospitals) and private providers (private midwife clinics, private obstetrician clinics, private clinics, and private hospitals [22, 24]. These services are organised into three tiers—primary care, secondary care, and tertiary care. Primary care comprises *puskesmas* and its network, private midwife clinics, private obstetrician clinics and private clinics; Private midwives are the largest private ANC providers and play a significant role in providing ANC services [24].

The EMTCT program, which was previously called the prevention of mother-to-child transmission (PMTCT) program, was introduced in 2005, and later updated in 2017 to include HIV and syphilis testing in ANC services. In 2018, hepatitis B testing was added, and the program was renamed the Triple Elimination Program (or EMTCT program). As with other MCH programs, financing for the EMTCT program comes from the central government (through *Anggaran Pendapatan dan Belanja Negara* (*APBN*)), or the sub-national governments at the provincial or district level (through *Anggaran Pendapatan dan Belanja Daerah* (*APBD*)). The national, provincial and district governments share responsibility for procuring the tests. The district government must forecast and plan for the number of test kits and reagents needed in their district for the subsequent year. While capacity-building activities for staff involved in the EMTCT program, such as training on testing and treatment, should ideally be funded at the district level, the provincial and national governments may also provide financial support for such activities [13]. Since the introduction of the national health insurance system (*Jaminan Kesehatan Nasional (JKN)*), the number of primary care facilities which provide ANC services and are registered with the JKN has grown rapidly [24]. However, from the latest available data, only 42% of private clinics, 60% of private hospitals, and 14% of private general practitioner clinics have joined the JKN [25].

### Research design and participants

This qualitative study was conducted between December 2019 and February 2020 in the Provinces of Bali and West Nusa Tenggara. These two provinces were selected to represent contexts of high (Bali) and low HIV (West Nusa Tenggara) testing rates at the time of the study. In 2022, there were 27,900 pregnant women tested for HIV and 16,407 tested for syphilis in Bali. In West Nusa Tenggara, the figures were 19,205 pregnant women tested for HIV and 10,110 tested for syphilis [26]. In Bali, there are nine districts or cities, made up of 717 villages. In 2022, almost 4.5 million people lived in the province, and 2.2 million of these were female [27]. West Nusa Tenggara province has ten districts or cities consisting of 1,151 villages. In 2022, an estimated 5.4 million people resided in this province and approximately half of these (2.7 million) were female [28]. A focus group discussion (FGD) and in-depth interviews were conducted with 25 health providers and policymakers. They were invited to participate in the study in December 2019. The FGD and interviews aimed to elicit the views and experiences of those responsible for designing and implementing the triple elimination program, including challenges they faced and strategies to address them. The FGD involved 12 staff from one Bali Provincial and five District Health Offices (DHO) working in the Disease Prevention and Control Division and the Family Health Division. In a decentralised health system like Indonesia’s, DHO and provincial health office (PHO) staff play an important role not only in implementing the policy and programs but also in program design and planning at the district and provincial levels.

In-depth interviews were then carried out in January and February 2020 with 18 health providers and policymakers from five districts in Bali province, along with two districts from West Nusa Tenggara Province. Participants for the FGD and in-depth interviews were selected purposively to include DHO staff, *puskesmas* staff, village midwives, and midwives from private clinics. We targeted policy makers with more than a year of experience in the delivery of Disease Prevention and Control Division and the Family Health Division program. For midwives and *puskesmas* staff, we targeted individuals with at least five ANC clients a month.

### Data collection

The FGD lasted two hours, and most interviews took about 45 minutes. The discussion and interviews were conducted by LPLW, DSM, DYK, DAMA, and KS, all with extensive experience in qualitative interviews. The FGD and interviews were informed by topic guides, covering questions about the implementation and operational aspects of the program. The details of the topics covered during the FGD and interviews are available in S1 Files 1, 2, and 3. All interviews and the FGD were conducted in Bahasa Indonesia, audio-recorded and transcribed verbatim. A formal written informed consent was obtained from all participants. LPLW, DSM, DYK, DAMA, and KS had access to information that could identify individual participants during or after data collection.

### Data analysis

WHO’s framework on the foundational requirements for the validation of EMTCT programs [14] guided the analysis. This framework was developed for use by the EMTCT validation secretariats, committees, and teams at national, regional, and global levels when assessing the minimum requirements of quality, availability, and accessibility of EMTCT information and services [14]. Based on the framework, our analysis considered four key domains: (1) laboratory quality; (2) data quality; (3) human rights, gender equality and community engagement; and (4) programmatic domain.

During the analysis, transcripts were coded to identify emerging themes across the four domains, with particular attention given to challenges in program implementation. Thematic analysis of the transcripts was undertaken [29] using the software NVivo 12 (QSR International Pty Ltd, Melbourne, Australia). To improve the quality of the data, findings were regularly discussed among the two leading researchers (LPLW and DSM) and the wider research team. Preliminary findings were fed back to participants to clarify particular points–a process known as ‘member checking’ [30].

This study was approved by the University of New South Wales (HC191010) and Udayana University (2981/UN14.2.2.VII.14/LP/2019) ethics committees. The Consolidated Criteria for Reporting Qualitative Research guidelines (COREQ) were used to guide comprehensive and rigorous reporting of the data collection procedures and the study findings [31].

## Results

Several themes pertaining to gaps in the program’s implementation emerged from the FGD and interviews. These themes, detailed below, are structured according to the WHO guidelines on the foundational requirements of EMTCT validation. A unique identifier was used to reference the citations illustrating key points. In total, we conducted one FGD with 12 policymakers, and 18 in depth interviews with policymakers and health providers. Among the 12 participants involved in the FGD, 11 of them were women, reflecting their higher representation of policymakers in maternal and child health programs. All participants involved in the in-depth interviews were female.

The following section describes the key themes emerging from the interviews and FGD. These themes were structured around the WHO’s framework on the foundational requirements for the validation of EMTCT programs and are visually represented in Table 1.

**Table 1:** Visual representation of the study findings.

Domains*	Themes	Example of quotes
Laboratory quality	Supply chain: Challenges and delays in the distribution of test reagents	*The district government has cut the funding*, *so we become under budget*. *I think this issue has been experienced by all districts [in this province]*. *In fact*, *it was discussed during the provincial meeting some time ago*. *The funding was lacking*, *so the test reagent was lacking*, *and we saw an interruption to the program*. *(interview_policymaker_dhokrs)*
Data quality	The complexity of reporting systems	*The problem is that the recording and reporting are too complicated*. *We need to put the records online*, *[need to] enter everything [to the online system]*. *First*, *[we] record [the data] manually [paper-based] and then [transfer it] to the computer; it will then be sent to the district*, *and finally*, *the district will verify it*. *I’m just surprised that we use [both] manuals [paper-based] and online*. *(interview_health provider_ktult)*
	Discrepancies in program coverage data from different divisions of the DHO	*Our midwife colleagues at the family health division have been collecting data on HIV tests from all facilities in the work area*. *In the disease prevention and control division*, *however*, *the data entered into the SIHA system is limited only to pregnant women who seek ANC in facilities that are trained in performing VCT*, *so for example*, *in xx district*, *only facilities with staff who had been trained on VCT or had access to SIHA at the puskesmas and hospitals can input the data to SIHA*, *so there will be a discrepancy in [the program coverage] data between the two divisions*. *(fgd_policymakers_stkhld)*
Human rights, gender equality and community engagement	Stigma	*That’s it; she won’t tell us about her HIV status…*. *We discussed this issue frequently in the midwife coordinator group; there were positive pregnant mothers in T [the other district] who did not return to the clinic*. *They were unable to be followed up there and came to our clinic without informing us of their HIV status*. *(interview_ policymaker_dhogia)*
Programmatic domain	Lack of training opportunities for staff	*The first is a problem related to staff capacity*. *The [capable] staff is often only available sometimes*. *Then*, *even if there are staff*, *they are not adequately trained [to carry out the test]*. *(interview_health provider_bpmlt)*
	Challenges in engaging with the private sector	*Some obstetricians are willing to be involved in this program*, *but not all of them*. *Some others still need to be [involved]*. *They seem unclear on the procedure to refer the women [to the puskesmas]*. *(interview_health provider_puskkrs)*

* Based on the WHO’s framework on the foundational requirements for the validation of EMTCT programs.

### Laboratory quality

#### Supply chain: Challenges and delays in the distribution of test reagents

During the interviews and FGD, participants from Bali Province told of experiencing stock-outs of test reagents. Due to budget limitations experienced by some MCH programs, or due to allocation of funding for other programs, funding for test reagent procurement had not been allocated by the provincial or district governments.

*The district government has cut the funding, so we become under budget. I think this issue has been experienced by all districts [in this province]. In fact, it was discussed during the provincial meeting some time ago*. *The funding was lacking, so the test reagent was lacking, and we saw an interruption to the program. (interview_policymaker_dhokrs)*

Almost all the districts in both provinces reported stock-outs of test reagents in early 2019. Participants mentioned however that not all tests were out of stock simultaneously. Of the three rapid test reagents used for HIV, syphilis, and hepatitis B screening, stock-outs were most frequent and for longer durations for syphilis and hepatitis B.

*For the triple E [elimination], we have three test reagents for those three screenings, right? Often, we only have the reagents for HIV, but not for hepatitis B or syphilis. This is a bit frustrating*. *If we say “triple“, those three [reagents] should have been available. (interview_policymaker_dhokrs)*

“*Like the other day*, *we ran out of syphilis and hepatitis B test reagents*, *so we can only do the HIV test …*.*the bottleneck is on the test reagents for syphilis and hepatitis B… not sure what happened; it [the problem] was at the national level*, *I guess*. *(interview_policymaker_dhobll)*

According to FGD participants, one district in Bali Province had never experienced stockouts of the three reagents and, in fact were able to test most women twice during their pregnancy (in the first and third trimesters) even though the regulation required women to be tested only once. They attributed this to the strong commitment of the head of the district to screening in pregnancy.

*We have an excellent example from J [name removed] district. All procurement was performed by the district using the health operational budget*. *The test is conducted not only once but twice in the first and third trimesters. It all depends on the commitment of the head of the district. (fgd_policymakers_stkhld)*

Also, during the FGD, one participant mentioned that delays in the distribution of reagents to the districts were often due to delays in the DHO requesting test reagents from the PHO.

*The reagents were available at the provincial level*. *However, to distribute it to the district, there needs to be a request from the district. If they [the district] don’t put a request, we can’t distribute it. (fgd_policymakers_stkhld)*

### Data quality

The EMTCT program activities, including numbers of women tested and their results, should be reported through existing surveillance systems (*Sistem Informasi HIV/AIDS & IMS* (SIHA) and *Sistem Informasi Hepatitis dan Penyakit Infeksi Saluran Pencernaan* (SIHEPI)). A collaboration between two *puskesmas*- and district-level divisions is required to perform this reporting mechanism. These are the family health division (which manages the maternal and child health program) and the disease prevention and control division (which manages both the surveillance systems).

#### The complexity of reporting systems

During the interviews, *Puskesmas* and DHO staff raised concerns about the complexity and time needed to record the data on women’s characteristics, risk behaviors, and testing on the voluntary and counselling (VCT) form, the sexually transmitted infection form, and the EMTCT form. Each form must first be completed in paper format and then entered into the computer-based surveillance systems.

*The problem is that the recording and reporting are too complicated*. *We need to put the records online, [need to] enter everything [to the online system]. First, [we] record [the data] manually [paper-based] and then [transfer it] to the computer; it will then be sent to the district, and finally, the district will verify it. I’m just surprised that we use [both] manuals [paper-based] and online. (interview_health provider_ktult)*

*The obstacle we face is time*. *We don’t have much time …. because there is too many [data]. We record everything; we enter it into the [paper-based] registration, enter it into the computer online, and then send it to the district health office. (interview_health provider_pmkkplt)*

#### Discrepancies in program coverage data from different divisions of the DHO

Concern was also raised about the discrepancies of testing coverage estimates for the three diseases reported by the two divisions involved in this reporting system. While both divisions use the number of pregnant women tested as the numerator, there was a difference in the denominator used to calculate program coverage. In the family health division, staff mentioned that data to assess the denominator should include all pregnant women in the districts, including those reported by village midwives through its local area monitoring or *pemantauan wilayah setempat—*which should ideally include women seeking ANC at all facilities, including private facilities. On the other hand, the disease prevention control division mentioned that the denominators included only those women who sought ANC from facilities with HIV VCT capacity or with access to the SIHA/SIHEPI reporting systems. This difference was perceived by the FGD participants to be problematic, particularly when evaluating testing coverage among pregnant women at the district level.

*Our midwife colleagues at the family health division have been collecting data on HIV tests from all facilities in the work area*. *In the disease prevention and control division, however, the data entered into the SIHA system is limited only to pregnant women who seek ANC in facilities that are trained in performing VCT, so for example, in xx district, only facilities with staff who had been trained on VCT or had access to SIHA at the puskesmas and hospitals can input the data to SIHA, so there will be a discrepancy in [the program coverage] data between the two divisions. (fgd_policymakers_stkhld)*

### Human rights, gender equality and community engagement

#### Stigma

Midwives revealed that women are often reluctant to be tested or followed up for treatment due to the stigma and discrimination associated with the diseases, particularly HIV.

*That’s it; she won’t tell us about her HIV status…. We discussed this issue frequently in the midwife coordinator group; there were positive pregnant mothers in T [the other district] who did not return to the clinic*. *They were unable to be followed up there and came to our clinic without informing us of their HIV status. (interview_policymaker_dhogia)*

*Some positive mothers did not want to start treatment*. *(fgd_policymakers_stkhld)*

*HIV-positive pregnant women are categorized as high risk, and the puskesmas will place the mother on their priority list of people who need to be visited. That is why they may feel embarrassed sometimes*; *neighbours may inquire why the health officer saw her frequently. (interview_policymaker_dhogia)*

In the FGD, participants reported that when *puskesmas* staff placed a mark in a woman’s pregnancy book to indicate a positive HIV status, it was not unusual for women to erase the mark from their book.

*The other issue is that we usually put a note in their ANC book when we find a positive pregnant mother*. *Some people recognized the mark and decided not to return. We did our best to work with HIV counsellors to persuade them, with some even hiding and attempting to conceal the mark in the ANC book*. *(fgd_policymakers_stkhld)*

Staff also reported that because of stigma, difficulties could arise in contacting women’s partners for notification and testing, especially where women who tested positive were fearful of communicating their status to their partners and family.

“*Please don’t tell my husband or mother-in-law”*, *she begged*. *We must keep it a secret because we want them to take their medicine and not miss their appointments*. *We had a client who believed her family was aware of her status and thus left the treatment*. *(interview_policymaker_dhogia)*

*She was also concerned about her husband’s HIV status and whether she contracted the virus from him*. *That is why she hides her HIV status from the rest of the family. We were unable to begin partner notification in this case. We will continue to monitor her condition without her husband’s knowledge. If the husband inquires, we will explain that the medication is for the baby’s health and contains vitamins. (fgd_policymakers_stkhld)*

### Programmatic domain

#### Lack of training for staff

The Indonesian EMTCT guidelines mention the need for trained staff to manage the program [13]. While participants from some districts in Bali stated they had received sufficient training to support program implementation, other participants from West Nusa Tenggara expressed frustrations around shortages in staff with the relevant training and skills to run the program.

*The first is a problem related to staff capacity. The [capable] staff is often only available sometimes*. *Then, even if there are staff, they are not adequately trained [to carry out the test]. (interview_health provider_bpmlt)*

Trained and experienced staff with the skills needed to run the program were frequently reassigned to other duties, resulting in a shortage of skilled personnel in the *puskesmas* in West Nusa Tenggara.

*Then the second problem is the staff turnover…*. *They [staff] are often moved [to other divisions], so the new team doesn’t know how to do it. (interview_health provider_pmkkplt)*

#### Challenges in engaging with the private sector

The MoH guidelines recommend all pregnant women seeking ANC services at *puskesmas* be screened free-of-charge for HIV, syphilis, and hepatitis B using rapid tests. On the other hand, those who seek ANC in the private sector, through private midwives, private obstetricians, or private hospitals, are to pay out-of-pocket for testing at those clinics or referred to a *puskesmas* for free HIV, syphilis, and hepatitis B screening [12]. Interviews with *puskesmas* staff from one district in Bali and stakeholders in the FGD revealed several challenges in engaging pregnant women from private providers because the providers did not perform the three tests for triple elimination or were unclear regarding the referral procedures.

*Some obstetricians are willing to be involved in this program, but not all of them. Some others still need to be [involved]*. *They seem unclear on the procedure to refer the women [to the puskesmas]. (interview_health provider_puskkrs)*

*Among those [pregnant women] who use JKN providers [the national health insurance], there is no big deal because the record from the pink [pregnancy] book is needed [by the providers] to claim a reimbursement*. *However, it becomes a problem when they do not use JKN. They don’t bother with pink books. (interview_health provider_pskbll)*

In the FGD, a participant from one district in Bali also mentioned that a private hospital in their area did not report the test results to the DHO through the surveillance system.

*One of the private hospitals in our district did procurement on their own*, *did the test in their clinics, but then did not report the activity to the SIHA. (fgd_policymakers_stkhld)*

## Discussion

The current study adds to the existing literature on the challenges of implementing Indonesia’s EMTCT program from the perspectives of policymakers, program implementors, and health providers. Our study demonstrates poor supply chain management of the tests resulting in frequent stock-outs. This issue has also been noted in other EMTCT studies in Malawi, South Africa and the United Republic of Tanzania [32]. A systematic review noted that among 13 studies from low and middle income countries that reported on the availability of point-of-care diagnostic testing for syphilis, malaria, HIV, hepatitis B virus, blood pressure, diabetes, and dyslipidaemia, 11 described frequent stock-outs of test kits [33]. Similar issue regarding the clinical supply stock-outs was also noted in a recent systematic review on barriers of HIV and syphilis rapid diagnostic tests in antenatal care settings in low and middle income countries [34]. This points to the importance of strong supply chain management when planning for the scale-up of EMTCT programmes. Further analysis of the data revealed that inaccurate forecasting and poor coordination between district and the provincial level health offices contributed to stock-outs, a finding consistent with several studies included in the above-mentioned systematic review [33] and additional studies from Uganda [35] and India [36]. This finding underlines the need for capacity building in stock forecasting and stock monitoring among staff at the district and provincial level. Our study also revealed that one district in Bali had a stronger capacity for stock management than the other districts. This was primarily due to the commitment of the district leader to prioritising funding for EMTCT logistics. Funding priorities in maternal health can differ substantially across districts, impacting the supply of key inputs to EMTCT programs. This phenomenon has also been observed in a previous study on immunisation programs in the country [37].

The high staff turnover rates identified by stakeholders in our study are consistent with the situation reported for EMTCT programs in rural Guatemala [38] and South Africa [39]. New staff often lack the skills and training to perform testing or to comply with government reporting requirements, contributing to fewer pregnant women being tested and inaccurate or missing data on testing coverage [38]. Since staff shortages and high staff turnover is common in Indonesia, especially in rural areas [40], there is an urgent need to provide ongoing training for staff and ensure that sustained funding is available to support these activities. The finding that West Nusa Tenggara experienced more frequent shortages and turnover of staff compared to Bali was probably because West Nusa Tenggara has more villages to serve and lower midwife- and nurse-to-population ratios compared with Bali [41]. It implies that ongoing staff training and funding are needed, particularly in areas where shortages exist.

Inadequate funding was also identified as a critical factor contributing to stock-outs. This is partly attributable to the different priorities of district governments. Under Indonesia’s decentralised health system, district authorities may take a different approach to funding and implementing national health policies [19, 42]. Compounding this problem is the absence of local policies to guide implementation by program managers at the district level [16]. Within one district, there may be significant variation in EMTCT implementation across ANC facilities including hospitals, *puskesmas*, or private practices, due to a lack of SOPs [16]. More effective engagement between national and local governments, private and public sector providers, and across ANC facility settings is therefore needed. This is important to better plan the allocation of funding for testing and referral. Developing local guidelines and clinic SOPs to support implementation of the MoH regulation on EMTCT, that reflect district priorities, also warrants exploration.

Private midwives have contributed significantly to ANC service delivery in Indonesia [43, 44]. The current study revealed, however, that the referral mechanism enabling pregnant women attending private ANC facilities to obtain triple EMTCT testing at the nearest public hospital or *puskesmas*, was not always functioning. This problem has been highlighted in an earlier study from Indonesia which showed that only 50% of women who sought ANC at a private facility took the HIV test after being referred to a *puskesmas* [45]. While private ANC facilities referred pregnant women to public health facilities for HIV testing, most of these women did not want to be tested [16]. Clear guidance for health care providers on referral processes is therefore needed to strengthen continuity of care and coordination between private and public ANC providers. Moreover, staff in private hospitals typically procured the test reagents themselves and may not have been aware of the requirement to report the number of women tested and the test results to the SIHA system, especially when the reagents were not provided by the DHO. This challenge of involving private hospitals in the reporting of the number of women tested and the testing results was possibly more common in Bali due to the higher concentration of private hospitals there (i.e. 65% in Java and Bali as opposed to 3.3% in Maluku, West and East Nusa Tenggara, Papua) [41].

Stigma and discrimination, in general, have typically hindered interventions around HIV testing in this study setting, whether targeted to pregnant women [46], clients of sex workers [47, 48], injecting drug users [49], or men who have sex with men [50]. The triple elimination program seeks to identify the infection status of pregnant women and their partners [14]. The current study has, however, observed that due to stigma, notifying partners in cases of women testing positive has its challenges, which have also been noted in other studies in Indonesia [51, 52] and Guatemala [38]. Another study in Papua New Guinea even noted the issue around loss to follow up which is particularly higher among women in a serodiscordant relationship [53]. Building staff competency through training is therefore essential to raising their confidence and skills especially in communicating test results to women and their partners. The problem of stigma, which persists in hampering HIV testing coverage, might potentially be countenanced in the future by building stronger partnerships with community organisations experienced in working closely with communities [54, 55].

This study has some limitations to consider. The research only took the views of health providers and policymakers. The intention was to explore the views and experiences of those implementing the EMTCT program in Indonesia, complementing earlier studies exploring the experiences of pregnant women in Indonesia on HIV and syphilis testing [21] and in other low and middle income countries [34]. Future interdisciplinary studies on challenges in implementing EMTCT program would benefit from jointly exploring the views of pregnant women, their partners, health cadres, and community organizations in the same study setting. Secondly, while efforts have been made to strengthen the validity of the data, for example, by member checking, the views and experiences of stakeholders who participated in the current study may or may not be generalisable beyond the local setting where this research was undertaken.

### Conclusion

This study provides new evidence on the barriers to effective implementation of HIV, syphilis, and hepatitis B testing at ANC facilities in Indonesia; and specifically on implementation of the EMTCT program. Stakeholders reported experiencing test reagent stock-outs which were particularly most frequent and for longer durations for syphilis and hepatitis B, inadequate skills in supply chain management, insufficient staff training to run the EMTCT program, high staff turnover, highly complex and time-consuming reporting systems, high levels of stigma that prevented women from being followed up and partners being notified, and inadequate reporting and referral of women from private providers to public ones for testing. Several potential responses to these challenges have been recommended, including capacity-building activities for staff in key areas such as stock forecasting and stock monitoring and ongoing training for inexperienced staff in managing the program, conducting the tests, reporting procedures, and counselling skills. Last but not least, a more effective partnership arrangement between national and local governments, between private and public sector providers, and between government and community organizations, including developing clear SOPs and guidelines to run the program, is critical.

## Supporting information

S1 Checklist Inclusivity in global research. (DOCX)

S1 File Focus group discussion guide. (DOCX)

S2 File In-depth interview guide among stakeholders in Bali. (DOCX)

S3 File In-depth interview guide among stakeholders in West Nusa Tenggara. (DOCX)
