# Supplementary material for: Challenges to integrating programs for the elimination of mother-to-child transmission of HIV, syphilis, and hepatitis B into antenatal care: Experiences from Indonesia
Source: PLOS Glob Public Health. 2024 Mar 6;4(3):e0002977. doi: 10.1371/journal.pgph.0002977 (PMC10917262; doi:10.1371/journal.pgph.0002977)
Supplement: S2 File — (DOCX) [file pgph.0002977.s003.docx]

**S2 File. In-depth Interview Guide Among Stakeholders in Bali**

**Questions:**

1. **Reasons for High Testing Rates:**
   - Why do you think your district has been able to achieve high testing rates among pregnant women for HIV, syphilis, and Hepatitis B? What factors have contributed to this success?
2. **Support Systems:**
   - Could you please elaborate on the type of support provided for HIV, syphilis, or hepatitis B testing in the ANC setting? This includes logistics, funding, technical support, and any other forms of assistance. If support has been provided, could you specify the sources and describe the different forms it takes? Has the level of support been adequate?
3. **Implementation challenges:**
   - What are the main challenges in implementing HIV, syphilis, or hepatitis B testing in the ANC setting?
     - Human Resources:
       - Are there adequate human resources for the EMTCT program? Why or why not?
     - Financing and Infrastructure:
       - Are there sufficient financial resources and facilities/infrastructure to support the program? Why or why not?
     - Organizational Coordination:
       - Is there effective coordination of the EMTCT program, both across divisions and among the field staff? Can you elaborate on this?
     - Health service delivery:
       - Have there been any challenges reported by pregnant women regarding the services related to HIV, syphilis, or hepatitis B testing? If so, could you provide some examples, please?
4. **Need for Further Support:**
   - What additional support is required to address these challenges?
